# Modeling the effect of delay strategy on transmission dynamics of HIV/AIDS disease

**DOI:** 10.1186/s13662-020-03116-8

**Published:** 2020-11-25

**Authors:** Ali Raza, Ali Ahmadian, Muhammad Rafiq, Soheil Salahshour, Muhammad Naveed, Massimiliano Ferrara, Atif Hassan Soori

**Affiliations:** 1 Department of Mathematics, National College of Business Administration and Economics, Lahore, Pakistan; 2 Institute of IR 4.0, The National University of Malaysia, 43600 UKM Bangi, Malaysia; 3 School of Mathematical Sciences, College of Science and Technology, Wenzhou-Kean University, Wenzhou, China; 4 Department of Mathematics, Faculty of Sciences, University of Central Punjab, Lahore, Pakistan; 5 Faculty of Engineering and Natural Sciences, Bahcesehir University, Istanbul, Turkey; 6 Department of Mathematics, Air University, Islamabad, Pakistan

**Keywords:** HIV/AIDS disease, Delay model, Stability analysis, Reproduction number, Computer results

## Abstract

In this manuscript, we investigate a nonlinear delayed model to study the dynamics of human-immunodeficiency-virus in the population. For analysis, we find the equilibria of a susceptible–infectious–immune system with a delay term. The well-established tools such as the Routh–Hurwitz criterion, Volterra–Lyapunov function, and Lasalle invariance principle are presented to investigate the stability of the model. The reproduction number and sensitivity of parameters are investigated. If the delay tactics are decreased, then the disease is endemic. On the other hand, if the delay tactics are increased then the disease is controlled in the population. The effect of the delay tactics with subpopulations is investigated. More precisely, all parameters are dependent on delay terms. In the end, to give the strength to a theoretical analysis of the model, a computer simulation is presented.

## Literature survey

HIV is the abbreviation of the human immunodeficiency virus. The white blood cells (WBCs) of the immune system are targeted and infected by HIV in humans. HIV produces millions of its copies in the bloodstream and consequently weakens the immune system by defeating WBCs. This type of WBCs is called CD4 cells or T-helper cells. HIV can be transmitted among people through different means, including unprotected sex with infected persons, usage of infected syringe for drug injection, and unsterilized surgical equipment [1]. Hence in order to prevent its transmission, we should spread awareness about protected sex and using sterilized medical equipment. HIV infected persons must go through antiretroviral treatment to fight infections. There is a way to treat HIV and no vaccine is available yet, but HAART is an effective technique used to slow down the progress of the disease. The Centre for Disease Control and Prevention (CDC) first recognized HIV in 1981. According to the CDC report of 2011, over 34 million people have died due to AIDS. According to the UNAID report of 2010, almost 36 million people are currently suffering from AIDS. In 2017, around 1.8 million people fell prey to HIV and around 0.94 million people died around the globe. By the end of 2018, almost 37.9 million people were suffering from HIV. In June 2019, almost 25.5 million people were reported to go through antiretroviral therapy. AIDS has now become a global issue in the 21st century. The mathematical models play a vital role in the study of the transmission dynamics of HIV/AIDS. The delay models are more compatible with the real-world as the dynamics of time from infection to infectiousness are captured by them. There are many models available in the literature, which exhibit the dynamics of this disease by the system of nonlinear differential equations without any delay, although the delay inclusion makes the model more realistic. The dynamical behavior of the population model with time delay has now become a hot topic of research [2]. Ogunlaran et al. [3] presented an effective strategy to fight against HIV infection in humans by using the compartment models. Duffin et al. [4] studied the dynamics of the immune deficiency virus of the complete course of infection. Omondi et al. [5] investigated the mathematical modeling of the impact of testing, treatment, and control of HIV transmission in Kenya. Wodarz et al. [6] designed the pathogenesis and treatment compartment in the modeling of HIV. Ida et al. [7] investigated nonlinear dynamical analysis of the deterministic model of HIV infection. Mastroberardino et al. [8] studied the dynamics of the virus in Cuba. Attaullah et al. [9] designed numerical schemes to study the dynamics of HIV infection in the human population. Theys et al. [10] studied the impact of HIV-I transmission dynamics in host evolution. Bozkurt et al. [11] investigated the stability analysis of nonlinear differential equations of the HIV epidemic model. Nosova et al. [12] planned a study of HIV-infection transmission and dynamics in the human population with the size of risk groups. Sun et al. [13] studied the estimation of the incidence rate of HIV with different methods of mathematical modeling. Sweilam et al. studied the modeling of HIV/AIDS and malaria disease with introducing the optimal control technique in the fractional order derivative [14]. Jawaz et al. presented a structure-preserving numerical method for the delayed modeling of the HIV/AIDS disease. In a biological sense, the numerical method keeps the structure-preserving properties like positivity, dynamical consistency, and stability. Mushanyu et al. investigated the impact of late diagnosis of HIV with the approach of mathematical modeling. The main focus of this article is to motivate individuals for self-testing, treatment, and awareness programs [15]. In 2020, Danane et al. investigated the fractional order model for hepatitis B virus infection with well-known assumptions of mathematics [16]. In 2020, Atangana et al. gave critical analysis about Covid-19 and how much facemasks are effective to control this pandemic around the world [17]. Goufo et al. has made great contributions and investigated the connection of HIV and Covid-19. Also, alert notes for some countries in the current strain of coronavirus were issued [18]. Atangana et al. investigated the dynamics of Ebola hemorrhagic fever in West African countries in [19]. Owusu et al. presented the dynamics of HIV model of Covid-19 with demographic effects by using the modeling with delay techniques of intracellular and interruptions [20]. Delayed mathematical modeling plays a significant role in the field of biomathematics. The current effort is presented for the modeling of HIV/AIDS disease by including the delay effect. The delay effect to control the epidemic of HIV/AIDS disease in the human population includes proper information and communication about disease, implementation of school-based sex education, motivation for voluntary counseling and testing, awareness programs organized in domestic level, focus on condom promotion and social marketing, motivation to sexually transmitted infection (STI) screening and testing, effective use of antiretroviral therapy, implementation of blood safety practices, and universal precautions. The structure of our paper based on the following sections. In Sect. 2, we discourse the HIV/AIDS model with a time delay effect and discuss the equilibria of the model. In Sect. 3, we investigate the reproduction number and the sensitivity of parameters of the model. In Sect. 4, we present well-known theorems for the local and global stability. In Sect. 5, we discuss computer results to strengthen a hypothetical analysis of the model. At the end, the conclusion of the study is presented.

## Model formulation

**Figure 1:**
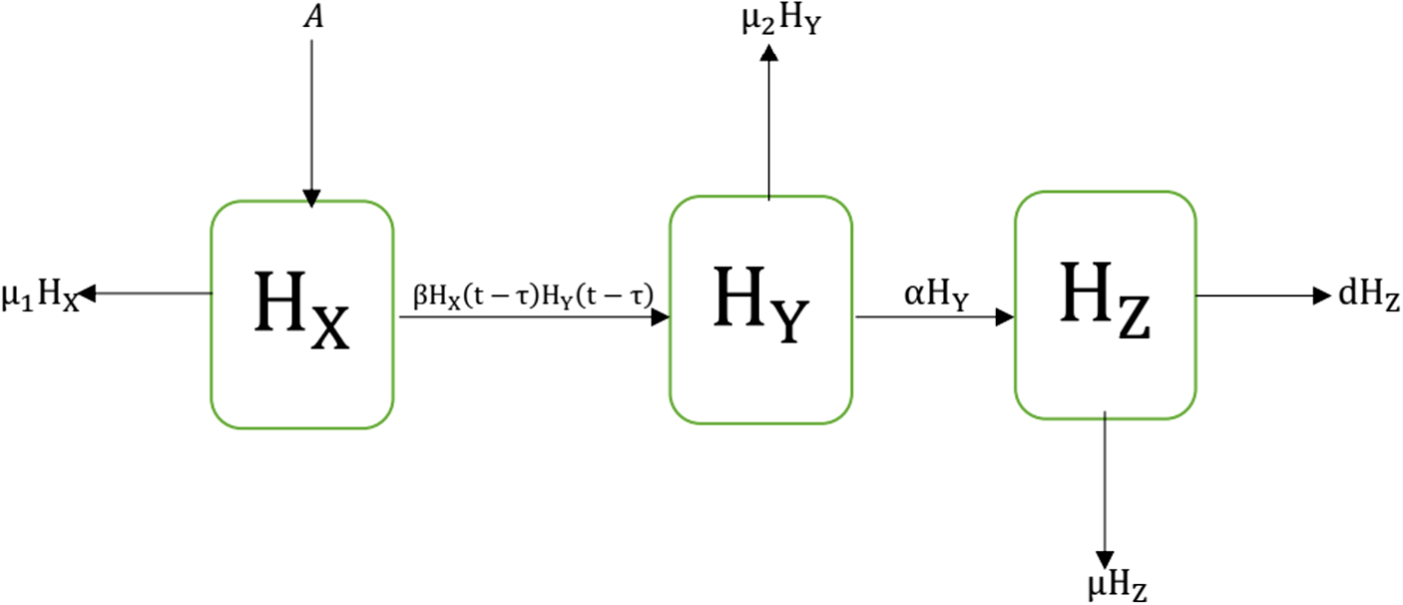
Stream map of the HIV/AIDS model

We consider the transmission of the HIV/AIDS epidemic model with a time delay effect in the human population. In our model, $N(t)$ represents the total population which we further categorize into the subpopulations as follows: At any time *t*, the uninfected/susceptible humans are denoted by $H_{X} (t)$, infectious humans are presented with $H_{Y} (t)$, while immune humans are presented with $H_{Z} (t)$. The transmission dynamics of the considered delay model as shown in Fig. 1.

The nonnegative constraints of the delay system are defined as follows: *μ* is the rate of natural incidences of immune humans, $\mu _{1}$ is the natural mortality rate of susceptible humans, $\mu _{2}$ is the natural mortality rate of infectious humans, *β* is the proportionality factor of the virus, *α* is the contact rate of infectious and immune humans, *d* is the death rate of humans due to virus which is greater than the natural death rate, *A* is the recruitment rate of humans. The following assumptions based on the modeling of HIV/AIDS with delay effect are as follows: the human population is homogeneous; considering only the contact of susceptible humans with infectious humans under the law of mass action, the latency period has been ignored. Without loss of generality, all other contacts with infectious humans have been overlooked. The mathematical representation of the HIV/AIDS disease is based on the following nonlinear delay differential equations:

2.1 $$\begin{aligned}& H_{X} ' =A-\beta H_{X} ( t-\tau ) H_{Y} ( t-\tau ) e^{-\mu \tau } - \mu _{1} H_{X} (t),\quad \forall t\in [- \tau ,0], \tau \in [0, \infty ), \end{aligned}$$

2.2 $$\begin{aligned}& H_{Y} ' =\beta H_{X} ( t-\tau ) H_{Y} ( t-\tau ) e^{-\mu \tau } -\alpha H_{Y} (t)- \mu _{2} H_{Y} (t),\quad \forall t\in [- \tau ,0], \tau \in [0,\infty ), \end{aligned}$$

2.3 $$\begin{aligned}& H_{Z} ' =\alpha H_{Y} (t)-(\mu +d) H_{Z} (t),\quad \forall t\in [ - \tau ,0 ], \end{aligned}$$

with the initial conditions $H_{X} ( 0 ) \geq 0$, $H_{Y} ( 0 ) \geq 0$, $H_{Z} ( 0 ) \geq 0$.

The total dynamics of system (2.1)–(2.3) is found by combining the first three equations as follows:

$$\begin{aligned}& H_{X} ' (t) + H_{Y} ' (t) + H_{Z} ' (t) \leq A - \mu _{1} N(t) \quad \mbox{and}\quad H_{X} (t) + H_{Y} (t) + H_{Z} (t) = N(t), \\& \frac{d N(t)}{dt} \leq A - \mu _{1} N(t). \end{aligned}$$

The feasible region of model (2.1)–(2.3) is as follows:

$$ \Gamma =\bigl\{ H_{X} ( t ), H_{Y} ( t ), H_{Z} ( t ) \in R_{+}^{3}: N(t) \leq A - \mu _{1} N(t) \bigr\} . $$

The initial value problem, $\phi ' (t) = A - \mu _{1} \phi (t)$, with $\phi ( 0 ) = N ( 0 )$, has solution $\phi ( t ) = k_{1} e^{- \mu _{1} \tau } - \frac{A}{\mu _{1}}$ and $\lim_{t\longrightarrow \infty } \phi ( t ) = \frac{A}{\mu _{1}}$. Therefore, $N ( t ) \leq \phi ( t )$, which shows that $\lim_{t\longrightarrow \infty } \sup N (t)\leq \frac{A}{\mu _{1}}$. Thus, all solutions of system (2.1)–(2.3) lie in Γ. The given region Γ is structure preserving for system (2.1)–(2.3), as desired. Hence, the region Γ is nonnegative invariant.

### Model equilibria

In this section, the system (2.1)–(2.3) will be shown to admit three types of equilibrium such as disease-free equilibrium (DFE), trivial equilibrium (TE), and endemic equilibrium (EE) as follows:

$$\begin{aligned}& A_{1} = \mathrm{Trivial}\ \mathrm{equilibrium}\ (\mathrm{TE}) = \bigl( H_{X}^{0}, H_{Y}^{0}, H_{Z}^{0} \bigr) =(0,0,0); \\& A_{2} = \mathrm{Disease}\mbox{-}\mathrm{free}\ \mathrm{equilibrium}\ ( \mathrm{DFE}) = \bigl( H_{X}^{1}, H_{Y}^{1}, H_{Z}^{1} \bigr) = \biggl( \frac{A}{\mu _{1}},0,0 \biggr); \\& A_{3} = \mathrm{Endemic}\ \mathrm{equilibrium}\ (\mathrm{EE}) = \bigl( H_{X}^{*}, H_{Y}^{*}, H_{Z}^{*} \bigr), \end{aligned}$$

where $H_{X}^{*} = \frac{\alpha + \mu _{2}}{\beta e^{-\mu \tau }}$, $H_{Y}^{*} = \frac{A- \mu _{1} H_{X}^{*}}{\beta H_{X}^{*} e^{-\mu \tau }}$, $H_{Z}^{*} = \frac{\alpha H_{Y}^{*}}{ ( \mu +d )}$.

## Reproduction number

In this section, we employ the next generation matrix method to the system (2.1)–(2.3), for obtaining the reproduction number by calculating the transition and transmission matrices as follows [21]:

$$ \begin{bmatrix} H_{Y}^{*}\\ H_{Z}^{*} \end{bmatrix} = \begin{bmatrix} \beta X e^{-\mu \tau } & 0\\ 0 & 0 \end{bmatrix} \begin{bmatrix} H_{Y}\\ H_{Z} \end{bmatrix} - \begin{bmatrix} \alpha + \mu _{2} & 0\\ -\alpha & \mu +d \end{bmatrix} \begin{bmatrix} H_{Y}\\ H_{Z} \end{bmatrix}. $$

Thus the transmission matrix *F* and transition matrix *V*, at the disease-free equilibrium (DFE) $A_{1}$, are

$$ F= \begin{bmatrix} \frac{\beta A e^{-\mu \tau }}{\mu _{1}} & 0\\ 0 & 0 \end{bmatrix}\quad \mbox{and} \quad V= \begin{bmatrix} \alpha + \mu _{2} & 0\\ -\alpha & \mu +d \end{bmatrix}. $$

Notice that the spectral radius of $F V^{-1}$ is called reproduction number and denoted as $R_{0}$, and in our case $R_{0} = \frac{\beta A}{\mu _{1} ( \alpha + \mu _{2} )} e^{-\mu \tau } $.

Before closing this section, we examine the sensitivity of the reproduction number with respect to each of the parameters involved. To that end, the following identities can be easily verified:

$$\begin{aligned}& S_{\beta } = \frac{\beta }{R_{o}} \times \frac{\partial R_{o}}{\partial \beta } =1, \qquad S_{A} = \frac{A}{R_{o}} \times \frac{\partial R_{o}}{\partial A} =1, \qquad S_{\mu _{1}} = \frac{\mu _{1}}{R_{o}} \times \frac{\partial R_{o}}{\partial \mu _{1}} =-1, \\& S_{\alpha } = \frac{\alpha }{R_{o}} \times \frac{\partial R_{o}}{\partial \alpha } =- \frac{\alpha }{\alpha + \mu _{2}},\qquad S_{\mu } = \frac{\mu _{2}}{R_{o}} \times \frac{\partial R_{o}}{\partial \mu _{2}} =- \frac{\mu _{2}}{\alpha + \mu _{2}}. \end{aligned}$$

Observe that the numbers $S_{\beta } $ and $S_{A}$ are positive. Meanwhile, the remaining numbers are negative. We conclude that the sensitive parameters of the reproduction number are *β* and *A*.

## Local stability

In this section, we present theorems to provide the properties of the equilibria of the model:

### Theorem

*The disease*-*free equilibrium* (*DFE*), $A_{2} = ( H_{X}^{1}, H_{Y}^{1}, H_{Z}^{1} ) = ( \frac{A}{\mu _{1}},0,0)$, *is locally asymptotically stable* (*LAS*) *if* $R_{0} <1$, *for any* $t \in [ - \tau ,0 ]$ *and* $\tau \in [0,\infty )$. *Otherwise the system* (2.1)*–*(2.3) *is unstable if* $R_{0} >1$.

### Proof

The Jacobian matrix for the system (2.1)–(2.3) at $A_{2}$ is evaluated as follows:

$$\begin{aligned}& J( A_{2} )= \begin{bmatrix} - \mu _{1} & - \frac{\beta A}{\mu _{1}} & 0\\ 0 & \frac{\beta A}{\mu _{1}} e^{-\mu \tau } -( \alpha + \mu _{2} ) & 0\\ 0 & \alpha & -(\mu +d) \end{bmatrix}, \\& \bigl\vert J( A_{2} )-\lambda I \bigr\vert = \begin{vmatrix} - \mu _{1} -\lambda & - \frac{\beta A}{\mu _{1}} & 0\\ 0 & \frac{\beta A}{\mu _{1}} e^{-\mu \tau } -( \alpha + \mu _{2} ) -\lambda & 0\\ 0 & \alpha & -(\mu +d) -\lambda \end{vmatrix} =0. \end{aligned}$$

Notice that all eigenvalues of the system are as follows:

$$ \lambda _{1} =- \mu _{1} < 0,\qquad \lambda _{2} =-(\mu +d)< 0, $$

but $\lambda _{3} =- ( 1- R_{0} ) <0$, if $R_{0} <1$.

Hence, all eigenvalues are negative and, by Routh–Hurwitz criterion, the given equilibrium $A_{2}$ is locally asymptotically stable.

If $R_{0} >1$, that is,

$$\begin{aligned}& \frac{\beta A}{\mu _{1} ( \alpha + \mu _{2} )} e^{-\mu \tau } >1, \\& \beta A e^{-\mu \tau } > \mu _{1} ( \alpha + \mu _{2} ), \\& - \mu _{1} ( \alpha + \mu _{2} ) +\beta A e^{-\mu \tau } >0, \end{aligned}$$

then $\lambda _{3} >0$. Hence, $A_{2}$ is unstable. □

### Theorem

*The endemic equilibrium* (*EE*), $A_{3} = ( H_{X}^{*}, H_{Y}^{*}, H_{Z}^{*} )$, *is locally asymptotically stable* (*LAS*) *if* $R_{0} >1$, *for all* $t \in [ - \tau ,0 ]$ *and* $\tau \in [0,\infty )$. *Otherwise*, *the system* (2.1)*–*(2.3) *is unstable if* $R_{0} <1$.

### Proof

The Jacobian matrix for the system (2.1)–(2.3) at $A_{3}$ is evaluated as follows:

$$ J ( A_{3} ) = \begin{bmatrix} -\beta H_{Y}^{*} e^{-\mu \tau } - \mu _{1} & -\beta H_{X}^{*} e^{-\mu \tau } & 0\\ \beta H_{Y}^{*} e^{-\mu \tau } & \beta H_{X}^{*} e^{-\mu \tau } -\alpha - \mu _{2} & 0\\ 0 & \alpha & - ( \mu +d ) \end{bmatrix}. $$

The eigenvalues of Jacobian matrix $J( A_{3} )$ are obtained as follows:

$$\begin{aligned}& \lambda _{1} =- ( \mu +d ) < 0, \\& \bigl\vert J ( A_{3} ) - \lambda I \bigr\vert = \begin{vmatrix} -\beta H_{Y}^{*} e^{-\mu \tau } - \mu _{1} -\lambda & -\beta H_{X}^{*} e^{-\mu \tau } \\ \beta H_{Y}^{*} e^{-\mu \tau } & \beta H_{X}^{*} e^{-\mu \tau } -\alpha - \mu _{2} -\lambda \end{vmatrix} =0, \\& \lambda ^{2} +\lambda [ C_{1} + \mu _{1} +2 \alpha + \mu _{2} - C_{2} ] +\bigl[ ( \alpha + \mu _{1} ) \bigl( ( \alpha + \mu _{2} ) - C_{2} \bigr) + C_{1} C_{2} \bigr]=0. \end{aligned}$$

Put, $C_{1} =\beta H_{Y}^{*} e^{-\mu \tau } $, $C_{2} =\beta H_{X}^{*} e^{-\mu \tau } $.

We check the conditions of the 2nd order Routh–Hurwitz criterion:

$$ C_{1} + \mu _{1} +2\alpha + \mu _{2} - C_{2} >0\quad \mbox{and}\quad ( \alpha + \mu _{1} ) \bigl( ( \alpha + \mu _{2} ) - C_{2} \bigr) + C_{1} C_{2} >0,\quad \mbox{if } R_{0} >1. $$

Hence, $A_{3}$ is locally asymptotically stable (LAS). □

## Global stability

In this section, we apply the well-known theorems to get the global properties of the equilibria of the model as follows [22].

### Definition 1

A function $V: R^{n} \rightarrow R$ is positive (negative) definite in a neighborhood of the equilibria of the model if $V ( 0,0,\dots ,0 ) =0$ and $V(x)> 0$ (<0) for $X\neq (0,0,\dots ,0)$ in Γ.

### Definition 2

A function $V: R^{n} \rightarrow R$ is positive (negative) semidefinite in a neighborhood of the equilibria of the model if $V ( 0,0,\dots ,0 ) =0$ and $V(x)\geq 0$ (≤0) for $X\neq (0,0,\dots ,0)$ in Γ.

### Theorem

*Suppose* $X ' =X ( H_{X} ( t ), H_{Y} ( t ), H_{Z} ( t ) )$ *has equilibria and there exists a feasible region “*Γ*” of the equilibrium points and a function* *V* *defined in* Γ *such that*:

i) *The first partial derivatives are continuous*;
ii) *V* *is positive definite*;
iii) $V '$ *is negative semidefinite*.

*Then the equilibria of the model are globally asymptotically stable* (*GAS*).

### Theorem

*The disease*-*free equilibrium* (*DFE*), $A_{2} = ( H_{X}^{1}, H_{Y}^{1}, H_{Z}^{1} ) = ( \frac{A}{\mu _{1}},0,0 )$, *is globally asymptotically stable* (*GAS*) *if* $R_{0} <1$, *for all* $t \in [ - \tau ,0 ]$ *and* $\tau \in [0,\infty )$. *Otherwise the system* (2.1)*–*(2.3) *is unstable if* $R_{0} >1$.

### Proof

Consider the Volterra–Lyapunov function $V: \Gamma \rightarrow R$ defined as [23]

$$\begin{aligned}& V= \biggl( H_{X} - H_{X}^{1} - H_{X}^{1} \log \frac{H_{X}^{1}}{H_{X}} \biggr) + H_{Y} + H_{Z},\quad \forall ( H_{X}, H_{Y}, H_{Z} )\in \Gamma , \\& \frac{dV}{dt} = \biggl( 1- \frac{H_{X}^{1}}{H_{X}} \biggr) \frac{d H_{X}}{dt} + \frac{d H_{Y}}{dt} + \frac{d H_{Z}}{dt}, \\& \frac{dV}{dt} = \biggl( \frac{H_{X} - H_{X}^{1}}{H_{X}} \biggr) \bigl( A-\beta H_{X} H_{Y} e^{-\mu \tau } - \mu _{1} H_{X} \bigr) +\beta H_{X} H_{Y} e^{-\mu \tau } - \mu _{2} H_{Y} -(\mu +d) H_{Z}, \\& \frac{dU}{dt} = \frac{- A ( H_{X} - H_{X}^{1} )^{2}}{H_{X} H_{X}^{1}} - \beta \bigl( H_{X} - H_{X}^{1} \bigr) \bigl( H_{Y} - H_{Y}^{1} \bigr) e^{-\mu \tau } \\& \hphantom{\frac{dU}{dt} ={}}{}- \mu _{2} H_{X}^{1} \biggl[1- \frac{\beta H_{Y} e^{-\mu \tau }}{\mu _{2}} \biggr]-(\mu +d) H_{Z} \end{aligned}$$

$\Rightarrow \frac{dU}{dt} \leq 0$ for $R_{0} <1$, and $\frac{dU}{dt} =0$ only if $H_{X} = H_{X}^{1}$, $H_{Y}^{1} = H_{Z}^{1} =0$. Therefore, the only trajectory of the system (2.1)–(2.3) on which $\frac{dU}{dt} =0$ is $A_{2}$. Hence, $A_{2}$ is globally asymptotically stable (GAS) in Γ. □

### Theorem

*The endemic equilibrium* (*EE*), $A_{3} = ( H_{X}^{*}, H_{Y}^{*}, H_{Z}^{*} )$, *is globally asymptotically stable* (*GAS*) *if* $R_{0} >1$, *for all* $t \in [ - \tau ,0 ]$ *and* $\tau \in [0,\infty )$. *Otherwise the system* (2.1)*–*(2.3) *is unstable if* $R_{0} <1$.

### Proof

Consider the Volterra–Lyapunov function $V: \Gamma \rightarrow R$ defined as

$$\begin{aligned} V =& K_{1} \biggl( H_{X} - H_{X}^{*} - H_{X}^{*} \log \frac{H_{X}}{H_{X}^{*}} \biggr) + K_{2} \biggl( H_{Y} - H_{Y}^{*} - H_{Y}^{*} \log \frac{H_{Y}}{H_{Y}^{*}} \biggr) \\ &{}+ K_{3} \biggl( H_{Z} - H_{Z}^{*} - H_{Z}^{*} \log \frac{H_{Z}}{H_{Z}^{*}} \biggr), \end{aligned}$$

where $K_{i}$ ($i=1,2,3$) are positive constants to be chosen later. Then

$$\begin{aligned}& \frac{dU}{dt} = K_{1} \biggl( 1- \frac{H_{X}^{*}}{H_{X}} \biggr) \frac{d H_{X}}{dt} + K_{2} \biggl(1- \frac{H_{Y}^{*}}{H_{Y}} \biggr) \frac{d H_{Y}}{dt} + K_{3} \biggl(1- \frac{H_{Z}^{*}}{H_{Z}} \biggr) \frac{d H_{Z}}{dt}, \\& \begin{aligned} \frac{dV}{dt} ={}& K_{1} \biggl( \frac{H_{X} - H_{X}^{*}}{H_{X}} \biggr) \bigl( A-\beta H_{X} H_{Y} e^{-\mu \tau } - \mu _{1} H_{X} \bigr) \\ &{}+ K_{2} \biggl( \frac{H_{Y} - H_{Y}^{*}}{H_{Y}} \biggr) \bigl( \beta H_{X} H_{Y} e^{-\mu \tau } -\alpha H_{Y} - \mu _{2} H_{Y} \bigr) \\ &{}+ K_{3} \biggl( \frac{H_{Z} - H_{Z}^{*}}{H_{Z}} \biggr) \bigl(\alpha H_{Y} -(\mu +d) H_{Z} \bigr), \end{aligned} \\& \begin{aligned} \frac{dV}{dt} ={}&{-} K_{1} \frac{A ( H_{X} - H_{X}^{*} )^{2}}{H_{X} H_{X}^{*}} - K_{1} \beta \bigl( H_{X} - H_{X}^{*} \bigr) \bigl( H_{Y} - H_{Y}^{*} \bigr) e^{-\mu \tau } \\ &{}- K_{2} \frac{\beta ( H_{X} - H_{X}^{*} ) ( H_{Y} - H_{Y}^{*} ) e^{-\mu \tau }}{H_{X} H_{Y}} - K_{3} \frac{ ( H_{Y} - H_{Y}^{*} ) ( H_{Z} - H_{Z}^{*} ) \alpha }{H_{Z}}. \end{aligned} \end{aligned}$$

For $K_{1} = K_{2} = K_{3} =1$, we have

$$\begin{aligned} \frac{dV}{dt} =&- \frac{A ( H_{X} - H_{X}^{*} )^{2}}{H_{X} H_{X}^{*}} -\beta \bigl( H_{X} - H_{X}^{*} \bigr) \bigl( H_{Y} - H_{Y}^{*} \bigr) e^{-\mu \tau } \\ &{}- \frac{\beta ( H_{X} - H_{X}^{*} ) ( H_{Y} - H_{Y}^{*} ) e^{-\mu \tau }}{H_{X} H_{Y}} - \frac{ ( H_{Y} - H_{Y}^{*} ) ( H_{Z} - H_{Z}^{*} ) \alpha }{H_{Z}} \leq 0 \end{aligned}$$

$\Rightarrow \frac{dV}{dt} \leq 0$ for $R_{0} >1$, and $\frac{dV}{dt} =0$ only if $H_{X} = H_{X}^{*}$, $H_{Y} = H_{Y}^{*}$, $H_{Z} = H_{Z}^{*}$. Therefore, the only trajectory of the system (2.1)–(2.3) on which $\frac{dV}{dt} =0$ is $A_{3}$. Hence, $A_{3}$ is globally asymptotically stable (GAS) in Γ by using the Lasalle’s invariance principle. □

## Computer results

In this section, we investigate the simulations of the system (2.1)–(2.3) by using different values of the parameters assumed and presented in Table 1.

**Table 1:** Values of parameters

Parameters	Values
*A*	0.5
*μ*	0.5
*d*	0.03
*β*	0.5 (DFE)
	0.7 (EE)
*α*	0.05
$\mu_{1}$	0.5
$\mu_{2}$	0.4

### Example 1

(Simulation at equilibria of the model without delay effect)

**Figure 2:**
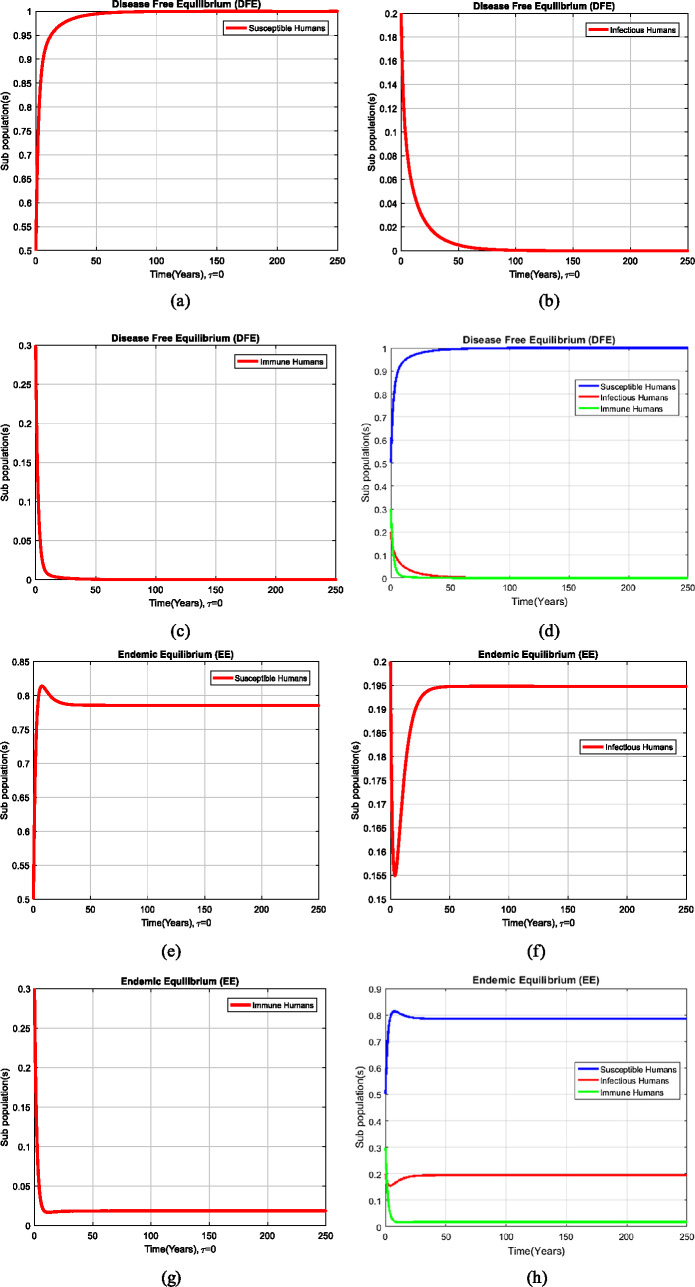
Graphs of the system (2.1)–(2.3) at the equilibria of the model

Figure 2(a)–(d) exhibits the solution of the system (2.1)–(2.3) at the disease-free equilibrium (DFE), $A_{2} = ( H_{X}^{1}, H_{Y}^{1}, H_{Z}^{1} ) = ( \frac{A}{\mu _{1}},0,0)$, by using the initial conditions $H_{X} ( 0 ) =0.5$, $H_{Y} ( 0 ) =0.3$, $H_{Z} ( 0 ) =0.2$, and the values of parameters presented in Table 1. Therefore, the system (2.1)–(2.3) converges to $A_{2}$, and the value of reproduction number in the absence of delay term is $R_{0} = 0.9091 < 1$. Moreover, Fig. 2(e)–(h) exhibits the solution of the system (2.1)–(2.3) at endemic equilibrium (EE) $A_{3} = ( H_{X}^{*}, H_{Y}^{*}, H_{Z}^{*} ) =(0.7857,0.1948,0.0184)$. So, the model converges to $A_{3}$, and the value of the reproduction number is $R_{0} = 1.2727 >1$, as desired.

### Example 2

(Simulation at endemic equilibrium of the model with time delay effect)

**Figure 3:**
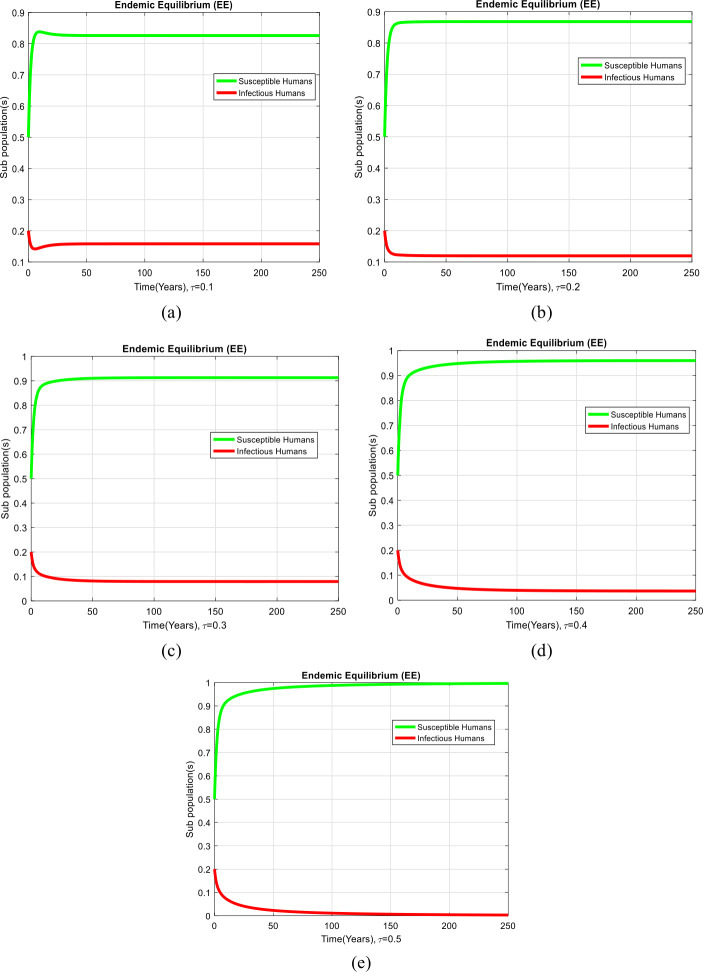
Graphs of the system (2.1)–(2.3) at the endemic equilibrium of the model with time delay effect

Figure 3(a)–(e), exhibits the solution of the system (2.1)–(2.3) at the endemic equilibrium (EE) with time delay effect. We can observe that the number of uninfected humans increases with the increase in the delay terms and ultimately the number of infected humans decreases. Eventually, the dynamics of the HIV/AIDS model moves to disease-free equilibrium with the effective use of delay tactics as observed in Fig. 3. Also, the reproduction number decreases with the increase in the delay tactics. Even more, in certain scenarios the value of the reproduction number is less than one. So, the dynamics of the reproduction number is independent of the values of the parameters.

### Example 3

(Effect of time delay term on the reproduction number)

**Figure 4:**
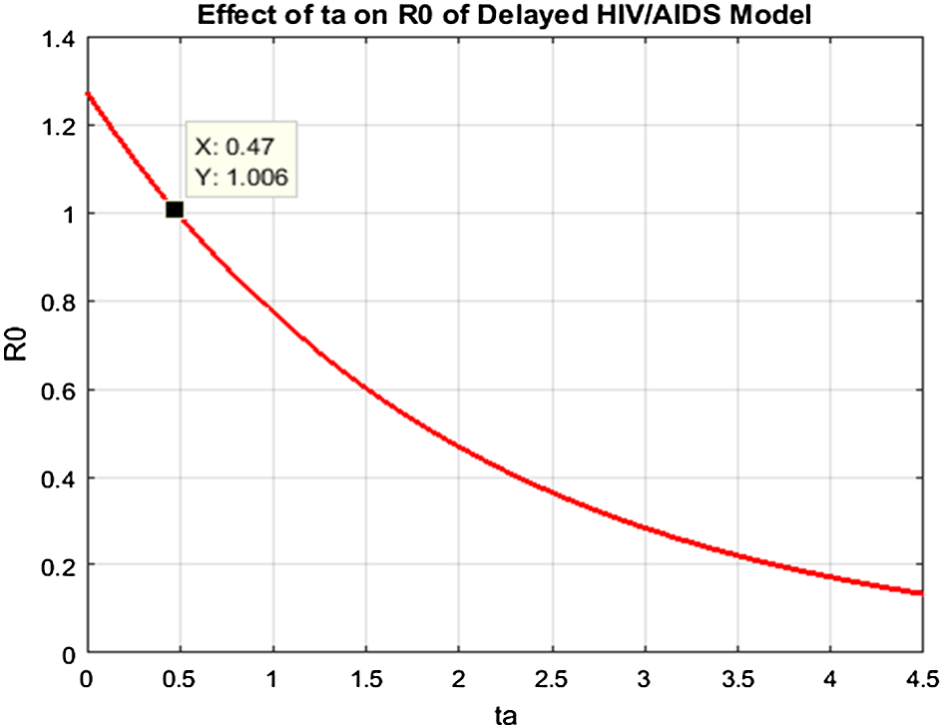
Comparison graph of the reproduction number with effect of the delay term of the model

Let $\tau =0.47$. It is clear that the reproduction value decreases, which moves the dynamics of the dynamical system from endemic to disease-free equilibrium. So, the absence of persistence of disease is stable. Yet, Fig. 4 displays the fact that the increases in delay strategy can overcome the epidemic of HIV/AIDS, as needed.

### Example 4

(Simulation for the effect of delay term on the infected component of the model)

**Figure 5:**
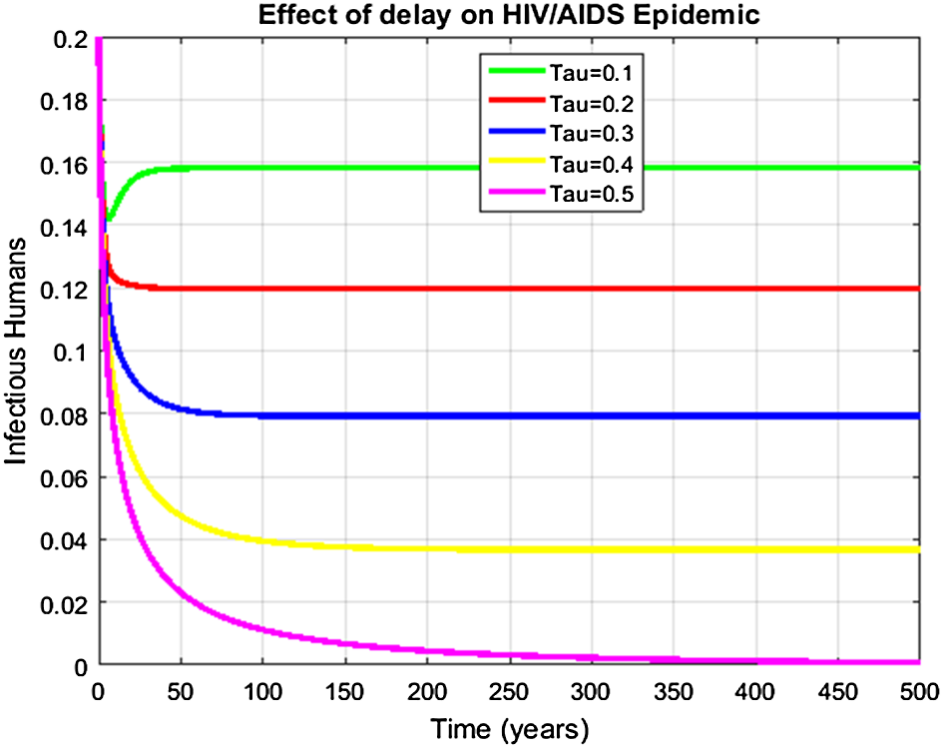
Display of the effect of delay term on infectious humans at the endemic equilibrium (EE) of the model

Letting *τ* take different values shows that the number of infectious humans tends towards and even touches zero. Ultimately, the described rate of infectious humans has been controlled at the given real data. Subsequently, Fig. 5 displays the fact that the delay strategy or delay tactics such as vaccination, quarantine, restrictions, and distancing measures, etc., have a vital role to control the epidemic of HIV/AIDS in the world, as desired.

## Conclusion

In the present study, we have investigated the dynamics of HIV/AIDS in humans with the strategy of delayed techniques. The whole population has been categorized into three components of the population, namely susceptible, infectious, and immune. We have verified the stability of the model locally and globally by using well-known theorems. Meanwhile, we have investigated the effect of delay techniques on the reproduction number and the infectious component of human population. After that, we have concluded that all the nonnegative constants of the model depend on the delay parameters. Furthermore, the delay techniques such as vaccination, antiretroviral therapy (ART), safe sex, and new gloves for every patient have been addressed. Before the end of that section, we have concluded that the analysis of delayed mathematical modeling plays a significant role in the dynamics of epidemic models. In the future, we shall propose the delayed fractional order model [24, 25]. Also, this idea could be extended for fractal–fractional and fractal–fractional partial differential equation models [26–28].

## Data Availability

This is not applicable for this article.
